# Dosimetry-Based Consideration on Remission and Relapse after Therapy with ^223^Ra-Dichloride in Castration-Resistant Prostate Cancer (CRPC) with Bone Metastases. A Case Report

**DOI:** 10.3390/diagnostics8010018

**Published:** 2018-02-27

**Authors:** Anna Maria Mangano, Massimiliano Pacilio, Pasquale Ialongo, Alessandro Semprebene, Guido Ventroni, Lucio Mango

**Affiliations:** 1 Nuclear Medicine Department, “S. Camillo-Forlanini” General Hospital, 00152 Rome, Italy; a.mangano@scf.gov.it (A.M.M.); a.semprebene@scf.gov.it (A.S.); g.ventroni@scf.gov.it (G.V.); 2 Medical Physics Unit, “Policlinico Umberto I” University Hospital, 00161 Rome, Italy; m.pacilio@policlinicoumberto1.it; 3 Radiology, “S. Camillo-Forlanini” General Hospital, 00152 Rome, Italy; p.ialongo@scf.gov.it

**Keywords:** prostate cancer, bone scan, bone metastasis, ^223^Ra-dichloride therapy

## Abstract

Here, we present the case of a 64-year-old male patient diagnosed with castration-resistant prostate cancer (CRPC) with bone metastasis, treated with abiraterone prednisone/prednisolone in combination with ^223^Ra-dichloride therapy, who had remission and a subsequent relapse of bone metastasis on repeated bone scans after therapy. We also discuss the possibility of continuing the ^223^Ra-dichloride therapy over the six planned administrations by administering other cycles at the same dose or at higher doses, if shown to be devoid of a significant increase in side effects, based on dosimetry considerations.

## 1. Introduction

In the advanced stages of many cancers, such as breast, lung and prostate carcinoma, metastatic bone disease is commonly seen [1]. The onset of symptoms associated with skeletal-related events (SREs), such as pain, fractures, hypercalcemia and medullar compression, is often due to the prostate cancer particular tendency to spread to the bone. These events result in significant morbidity, worsen patient quality of life and comprise the most frequent cause of death in these patients. The onset of SREs is delayed to some extent by pharmacological palliative treatment for bone pain [2]. Bone-targeting radiopharmaceuticals have also been included among available treatment options, because they have been shown to be efficient at metastatic bone pain relieving, particularly in patients with widespread bone disease [3,4,5].

^223^Ra-dichloride is a novel bone-seeking calcium mimetic alpha emitter, accumulating in areas of increased bone turnover that is being developed to target metastatic bone disease. A greater biological effectiveness compared to beta radiation is due to the high linear energy transfer (LET) of alpha radiation, causing the rise of cytotoxicity which is independent of dose rate, cell cycle growth phase, and oxygen concentration [6,7]. During the last years, the safety and efficacy of palliation of painful bone metastasis in patients with castration-resistant prostate cancer (CRPC) using ^223^Ra-dichloride (Xofigo^®^; Bayer HealthCare, Berlin, Germany) has been shown in many clinical trials. ^223^RaCl_2_ is becoming a new standard of care for patients with CRPC and bone metastasis due to significantly improved overall survival and very low toxicity [8,9,10,11,12,13,14,15,16]. An international randomized controlled trial, including 921 patients from over 100 treatment centers located in 19 countries, [17] highlighted an increase in life expectancy in patients treated with ^223^RaCl_2_ than that of patients in the placebo group, with overall survival of 14.9 months compared to 11.3 months. Therefore, ^223^Ra-dichloride had a significant survival benefit, delaying the onset of SREs with an acceptable safety profile. This effect was not previously registered for other available radiopharmaceuticals [18]. These results indicate that ^223^Ra-dichloride can be used as a systemic treatment strategy for patients with CRPC who develop bone metastasis, both as first-line and as rescue therapy. The use of ^223^Ra-dichloride has been approved for patients with CRPC who have symptomatic bone metastasis without visceral disease, whether they were treated or not with docetaxel. It was also reported by some authors that patients treated with ^223^Ra-dichloride and a concomitant bone-targeting agent (BTA) appeared to have longer time to first SREs than those treated without a concomitant BTA [19]. The same group of authors also says that there are changes in alkaline phosphatase (ALP) dynamics and overall survival (OS) in metastatic castration-resistant prostate cancer (mCRPC) patients treated with ^223^Ra: ALP decline was associated with longer OS and time to first SREs [20].

## 2. Case Report

A 64-year-old male patient, with no history of concomitant diseases, had on May 2007, PSA levels of 6.0 ng/mL (normal range, <4 ng/mL). On June 2007, he was diagnosed with Gleason 6 [21] prostate cancer by echography and transrectal biopsy. A successful radical prostatectomy with obturator bilateral lymphadenectomy was performed in September 2009. At that time, the patient had PSA levels of 9.7 ng/mL. The histopathology report of formalin-fixed sections revealed Gleason grade 7 right lobe adenocarcinoma without extraprostatic invasion, non-affected surgical margins and negative lymphatic nodes (pT2aN0) [22]. 

A prostate bed Radiotherapy (RT) treatment (66 Gy in 28 daily fractions) was performed on July 2011 because of an increase in PSA value (= 15.0 ng/mL), but PET/CT choline was negative. 

In November 2012, a PET/CT choline showed high concentration of the tracer in the right humeral region, in the absence of corresponding CT alterations. In that period, a treatment with Casodex 150 mg + Nolvadex 20 mg/day + decapeptyl 3.75 mg (1 vial/month since March 2013) was started.

In April 2015, PSA levels were 21.5 ng/mL and a bone scan revealed the presence of a lesion on the right humeral neck and head. A single session RT to relieve pain was performed on the right shoulder.

**Figure 1:**
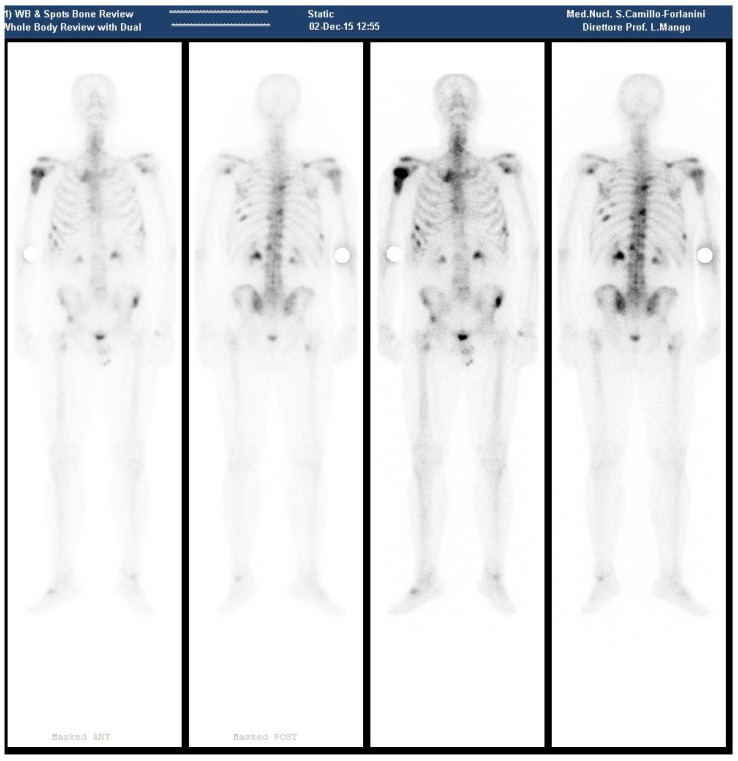
(December 2015—Bone scan pre ^223^RaCl_2_ Therapy, showing several metastatic sites as described in the text).

In December of the same year, the patient presented at our department. A new bone scan revealed hyperaccumulation on the proximal epiphysis and diaphysis of right humerus; left scapular glenoid; upper margin right scapula; rear bow of XII, IX, VI right rib, IX, VIII, VI left rib, front bow VI and VII right rib, IV and VII left ribs; D6, D9, D10, D12, L1, L2 vertebral body; anterior superior iliac spines; upper corner of the right acetabular roof; left sacroiliac synchondrosis; right femoral pertrocanteric region (Figure 1).

In January 2016, PSA levels went up to 317.35 ng/mL and a Total Body CT was negative for visceral disease. We started a therapy with abiraterone prednisone/prednisolone in combination with ^223^Ra-dichloride therapy (six administrations of 55 kBq per kg i.v., once every four weeks). The last cycle of administration of ^223^Ra-dichloride was on 30 May 2016, while Prednisone 5 mg + Abiraterone acetate 250 mg were continued.

**Figure 2:**
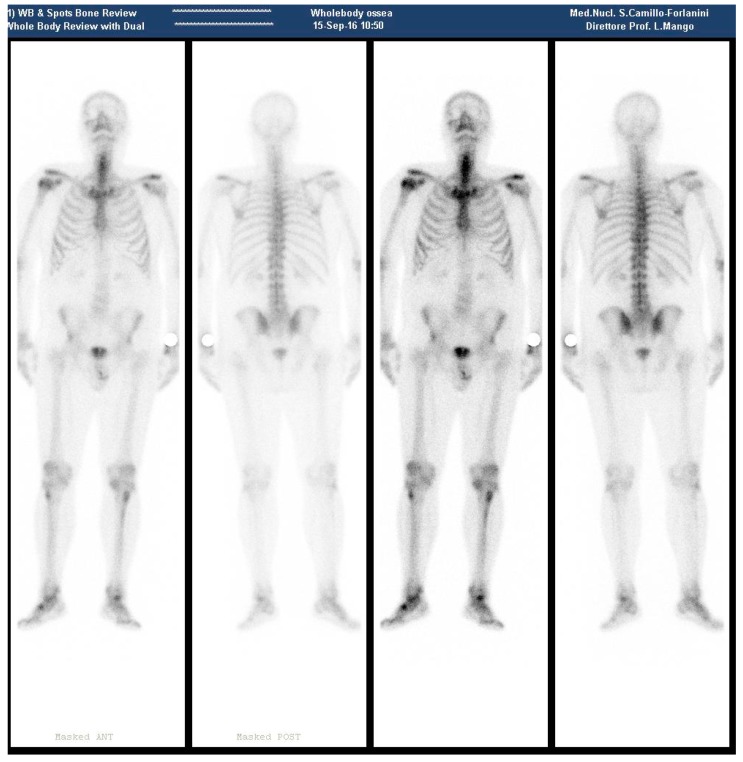
(September 2016—Bone scan 4 months after ^223^RaCl_2_ last cycle, with no metastatic site, except for the surgical neck of the right humerus).

During September 2016, a negative bone scan was obtained, except for a mild tracer accumulation on the surgical neck of the right humerus (Figure 2). 

In January 2017, the patient presented mild shoulder pain, but did not take pain medication. Total Body CT was negative for visceral disease, with a PSA level of 6.41 ng/mL. 

**Figure 3:**
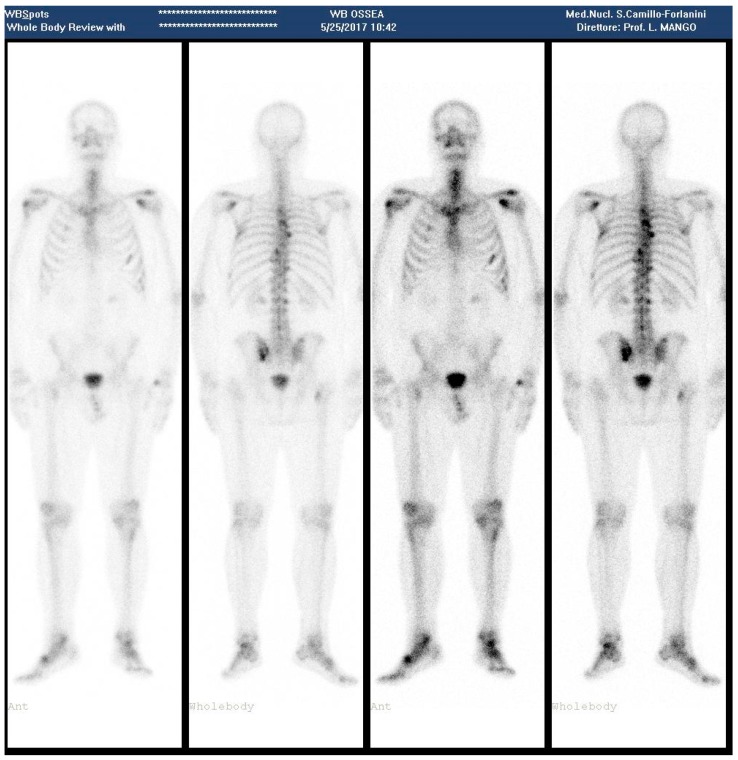
(May 2017—Bone scan 1 year after the last ^223^RaCl_2_ cycle, showing appearance of new metastatic sites).

In April, PSA levels elevated to 23.95 ng/mL and in May to 47.23 ng/mL, and a bone scan revealed vertebral hyperconcentration in D3, D5; left-cost-vertebral articulation of D8 at level of the right iliac crest; slight increase of activity in the left sacro-iliac region (Figure 3). 

Considering the Progression of Disease (PD), chemotherapy began with Docetaxel.

## 3. Discussion

It has been shown by a large multicentric double-blinded randomized phase III clinical study, that siz administrations of 50 kBq/kg of ^223^RaCl_2,_ administered once every four weeks, resulted in a significant decrease in bone marker levels and a significant delay in the onset of SREs, resulting in an improved OS and pain relief [23]. The administered ^223^RaCl_2_ activities depend on body weight and are considerably lower than those of beta-emitting radiopharmaceuticals due to their higher biological effectiveness [7].

In the present case, bone pain relief and significant decrease in PSA levels were obtained with the registered schedule of six injections of ^223^Ra-dichloride (55 kBq/kg). Moreover, four months after the administration of the final dose, the bone scan showed improvements of the metastatic burden, with tracer uptake quite normal in most of the metastatic sites observed before therapy. However, PD was indicated by the appearance of new metastatic lesions, as also shown by other authors [24].

The patient did not show major toxicities during or after the therapy. In a previous work [25], we assessed the utility of SPECT/CT for dosimetry determination in Targeted Radionuclide Therapy, and that with appropriate programs fixed therapy dose administration of β-emitters radionuclide can be implemented with no enhanced side effects [5]. Using the standard administration schedule, treatments with ^223^RaCl_2_ are associated with low rates of both hematological and non-hematological adverse events. On this basis, the possibility of dose escalation for treatments with ^223^RaCl_2_ is also under consideration [26].

Radiation-induced myelotoxicity remains a major concern, particularly for dose-escalation projects. It has been demonstrated that no grade 4 toxicities, and infrequent grade 3 toxicities have been observed in past studies [8,9]. Microdosimetry could help understand these outcomes. The percentage of cells that received a potentially toxic absorbed dose (2 or 4 Gy) as a function of the average absorbed dose over the marrow cavity, was determined in a previous study, which showed that: (1) the cellular-absorbed dose has a heterogeneous distribution strongly dependent on the position of the cell within the marrow cavity; (2) increasing the average marrow cavity-absorbed dose (by increasing the administered activity) results in only a small increase in potential marrow toxicity (i.e., the number of cells receiving more than 2 or 4 Gy) for a range of average marrow cavity-absorbed doses from 1 to 20 Gy [27]. 

In conclusion, the recovery of bone metastatic disease in areas other than those treated with ^223^RaCl_2_, and the absence of significant side effects, suggest the possibility of continuing the Xofigo therapy. This can be done by administering further cycles at the same dosage, or at higher dosages. The proper dosage could be outlined before therapeutic administrations, elaborating a dosimetric methodology which provides patient-specific absorbed dose to lesions, customizing the treatment on the individual patient considering one (or more) selected lesions [26,28]. A good level of correlation between ^99m^Tc-diphosphonate and ^223^Ra-dichloride percent uptake was observed [26], demonstrating that ^99m^Tc-MDP images provide a good scintigraphic description of lesional uptake for ^223^RaCl_2_ therapy. So, lesion dosimetry could be performed easily using the first ^223^RaCl_2_ administration as if it were a tracer administration, overcoming the problem of the low ^223^Ra image quality by the use of the conventional WB scan performed before the therapy. Therefore, macrodosimetry of bone lesion is feasible, as already widely demonstrated [26,28,29]. However, more efforts are needed to obtain a dose-response correlation in terms of bone lesion control (local response), providing also dosimetry endpoints useful to design tailored dosimetry-guided treatments in ^223^Ra-therapy [30].
